# Help at Hand, Brother

**Published:** 1886-10-02

**Authors:** 


### Help at Hand, Brother.

**THE HOSPITAL EMPLOYES' EMPLOYMENT COLUMN.**

*The Hospital* makes no charge for advertisements of the following classes seeking situations, provided each advertisement is accompanied by the Employment Coupon, which will be found affixed to one of the advertisement pages of each number of *The Hospital*:—Persons employed by the governors of hospitals and infirmaries, asylums, nursing and convalescent institutions,. Space for which no charge is made, is limited to twenty-four words.

Additional space is charged for at the rate of 1*s.* per line of eight words. Employers advertising for help are charged 1*s.* per line.

**THE IN-PATIENTS' EMPLOYMENT COLUMN.**

In order to place within the reach of our readers an advantage offered by no other Journal, it has been determined, for the present, to retain an absolutely

**FREE ADVERTISEMENT COLUMN**

for the assistance of patients on their discharge from the hospitals; the hardness of whose lot is graphically described in the narrative entitled "Discharged," by George Fleming [see *The Hospital*, vol. i, pp. 4 and 5], provided each advertisement is accompanied by the Employment Coupon, which will be found among the advertisement pages of each number of *The Hospital.*

**The Coupon entitles the holder** to one advertisement of not more than 24 words, subject to the following conditions:

Each advertisement must have the full name and address of the advertiser affixed, together with the name of the hospital and of the ward in which the patient is located at the date of his application. Each statement concerning a patient must be verified by the signature of the chaplain, the sister or nurse of the ward, or by that of one of the chief officials at the hospital. Patients are invited to state any circumstances which make their cases specially distressing or deserving, but all such statements must be verified by the hospital officials or by the minister of religion of the place of worship which the person referred to habitually attends. **For the present these free patients' employment advertisements are confined to in-patients only.**

**Each advertisement and statement** must be made out separately and be distinctly written on one side of the paper only.

**All letters** containing these advertisements must be endorsed on the outside of the envelope, "Free Advertisement Department," and must contain the full name and address of the advertiser for the Editor's information, in cases where a *nom de plume* is employed by the advertiser. The Editor reserves to himself the right of refusing an advertisement without any reason whatever being given for so doing, and takes no responsibility with regard to any engagements arranged through this column.

For insertion in the current week's issue, copy for this column must reach us by noon on Wednesday.

